# Tumor Mutation Burden Prediction Model in Egyptian Breast Cancer patients based on Next Generation Sequencing

**DOI:** 10.31557/APJCP.2021.22.7.2053

**Published:** 2021-07

**Authors:** Auhood Nassar, Ahmed M Lymona, Mai M Lotfy, Amira Salah El-Din Youssef, Marwa Mohanad, Tamer M Manie, Mina M G Youssef, Iman G Farahat, Abdel-Rahman N Zekri

**Affiliations:** 1 *Department of Cancer Biology, National Cancer Institute, Cairo University, Cairo, Egypt. *; 2 *Department of Surgical Oncology, National Cancer Institute, Cairo University, Cairo, Egypt. *; 3 *Department of Biochemistry, College of Pharmacy, Misr University for Science and Technology, Cairo University, Cairo, Egypt. *; 4 *Norfolk and Norwich University Hospital, Norwich, UK. *; 5 *Department of Pathology, National Cancer Institute, Cairo University, Cairo, Egypt. *

**Keywords:** breast cancer, tumor mutation burden, ER- PR- HER-2, Ki-67

## Abstract

**Objectives:**

This study aimed to identify the tumor mutation burden (TMB) value in Egyptian breast cancer (BC) patients. Moreover, to find the best TMB prediction model based on the expression of estrogen (ER), progesterone (PR), human epidermal growth factor receptor 2 (HER-2), and proliferation index Ki-67.

**Methods:**

The Ion AmpliSeq Comprehensive Cancer Panel was used to determine TMB value of 58 Egyptian BC tumor tissues. Different machine learning models were used to select the optimal classification model for prediction of TMB level according to patient’s receptor status.

**Results:**

The measured TMB value was between 0 and 8.12/Mb. Positive expression of ER and PR was significantly associated with TMB ≤ 1.25 [(OR =0.35, 95% CI: 0.04–2.98), (OR = 0.17, 95% CI= 0.02-0.44)] respectively. Ki-67 expression positive was significantly associated with TMB >1.25 than those who were Ki-67 expression negative (OR = 9.33, 95% CI= 2.07-42.18). However, no significant differences were observed between HER2 positive and HER2 negative groups. The optimized logistic regression model was TMB = -27.5 -1.82 ER – 0.73 PR + 0.826 HER2 + 2.08 Ki-67.

**Conclusion:**

Our findings revealed that TMB value can be predicted based on the expression level of ER, PR, HER-2, and Ki-67.

## Introduction

BC is a heterogeneous disease comprises different subtypes classified according to the expression of ER, PR, HER-2, and Ki-67 [Caswell and Swanton, 2017; Osako et al., 2017). Most cases are with Hormone-receptor positive tumors and have a relatively better clinical outcome (Dunnwald et al., 2007). By contrast, triple negative BC is an aggressive and heterogeneous disease with poor prognosis, which increases the need for other treatment modalities like immunotherapy (Davis et al., 2014). Several studies revealed the role of the immune system in cancer; including cancer immunoediting process which eliminates immunogenic tumor cells by the host immune system (Criscitiello and Curigliano, 2015). 

Cancer, as a genetic disease, results in accumulation of somatic mutations in the DNA of the affected cells. Some somatic mutations can give rise to neoantigens that are recognized and targeted by the immune system. Not only immunogenic neoantigens affect the ability of T cells to identify and kill tumor cell, but mutations in immunologically relevant genes can also do, for example; mutations in JAK1, JAK2, B2M, and STK11genes (Skoulidis et al., 2018). 

TMB represents one of the most rapidly emerging biomarkers for cancer immunotherapy and many ongoing clinical trials registered in ClinicalTrials.gov are using it as a stratification biomarker (Chan et al., 2018). Currently, immune checkpoint blockade (ICB) can offer a significant clinical benefit to cancer patients; most notably melanoma (Hodi et al., 2010), non-small-cell lung cancer (NSCLC) (Borghaei wt al., 2015), urothelial cancer (Rosenberg et al., 2016) and renal cell carcinoma (Motzer et al., 2018). 

There are limited reports about TMB in BC. However, TMB is higher in ER-negative tumors when compared to ER-positive tumors, and it may be of a relatively high value in TNBC patients (Luen et al., 2016). The reference for TMB quantification is Whole Exome Sequencing (WES). However, it was reported that gene panels that target 1.5 to 3 Mb of the genome space are precise for TMB estimation (Buchhalter et al., 2019). In our previously published data, we reported the most identified somatic mutations in the first Egyptian cohort to sequence a multiple-gene panel of cancer related genes on BC patients (Nassar et al., 2020). Here in, using the same panel, we aims to identify the TMB value in Egyptian BC patients and to find the best model to predict it, based on the expression of ER, PR, HER-2, and Ki-67. Thereby, predicting patients’ prognosis and response to treatment. 

## Materials and Methods

*Patients*

Fifty-eight female Egyptian BC patients were enrolled in this study from the Egyptian National Cancer Institute. They were all naive to treatment and their classification was according to age, histological grade, lymph node involvement, menopause status, ER, PR, HER-2, and Ki-67 expression. All the patient’s clinicopathological features were obtained from clinical records. All the tissue samples were preserved at −80 °C until being extracted.

*Ethical approval*

Fifty-eight tissue samples were collected at surgery and after the agreement of all patients with a written informed consent. This study was approved by the Institutional Review Board of NCI, Cairo University, Egypt which conducted in accordance with ICH-GCP guidelines (IRB number: IRB00004025). 

*Targeted DNA library preparation and sequencing with the ion torrent proton system*

The genomic DNA was isolated using QIAamp® DNA Mini Kit (Cat. No. 51304, Qiagen, Germany) following manufacturer’s instructions. DNA was then measured using Qubit® 3.0 Fluorometer (Cat. No, Q33216, Thermo fisher Scientific Inc, USA). The library was constructed using the Ion AmpliSeq Comprehensive Cancer panels (Ion AmpliSeq CCP, Life Technologies, Cat. No. 4477685) and the Ion AmpliSeqTM Library Kit 2.0 (Cat. No.4480441). Libraries were quantified using the Ion Library Quantitation Kit (Life Technologies, Cat. no. 4468802) and sequenced with the ion torrent proton system.

*Detection of somatic mutations and calculation of TMB value*

The high-quality reads of each NGS run were aligned to the human reference genome (version hg19) using the Torrent Suite. TSVC Torrent Suite module was used to call the somatic variants with hotspots. Then, the variants with all available public population information were annotated using ANNOVAR (Yang and Wang, 2015) package. The Catalogue of Somatic Mutations in Cancer COSMIC (Tate et al., 2019) and CIViC (Griffith et al., 2017) databases were used to annotate variants in cancer. TMB was defined as the number of somatic, non-silent, base substitution, and indel mutations per mega base of the examined genome.

*Statistical analysis*

All data were analyzed with SPSS (version 22.0). According to the quartile method, method divides dataset into three points (a lower quartile, median, and upper quartile) to form four groups, the TMB distribution was divided into 4 groups as follow: A (0.00-0.625), B (0.626-1.25), C (1.251-2.50), and D (>2.50). Forest plot for univariate logistic regression analysis was used for TMB distribution according to ER, PR, HER-2, & Ki-67 expression. 

*Machine learning classification models for prediction of TMB in the studied BC patients*

Different classification machine learning algorithms; logistic regression, kernel support vector machine (SVM), K-nearest neighbor (KNN), Decision tree and Forrest decision, were initially evaluated to select the optimal classification model for prediction of TMB level according to patient’s receptor status. Grid search was used to find the best hyperparameters for the highest accuracy across an extent of model specific parameters. First, the dataset was split into 75% training set and 25% test set. The models were built in 6-fold cross validation (CV) on the training set to evaluate the model on different validation sets. The model performance was evaluated as accuracy, Receiver Operating Characteristic (ROC) curve with area under the curve score (AUC) and Matthews Correlation Coefficient. The evaluation metric generally regarded as a balanced measure that represents, in a single value, the confusion matrix of the classifier even if the classes are of very different sizes. The MCC value is between -1 and +1 where +1 indicates the perfect prediction, 0 an average random classification prediction and -1 the perfect inverse prediction. 

## Results

*Patients’ clinicopathological characteristics*

The median age of the studied patients was 54.6 (range from 29 to 77). The majority of the presented patients were with ER-positive (65.5%), PR-positive (55.2%), HER2-negative (70.7%), and Ki-67 positive (56.9%). Also, 41 patients out of 58 (70.7%) were postmenopausal; 48 patients out of 58 (82.7%) had positive lymph node involvement and only 9 patients out 58 (15.5%) were classified as triple negative (Table 1). 

Distribution of TMB in our studied sample set

**Figure 1:**
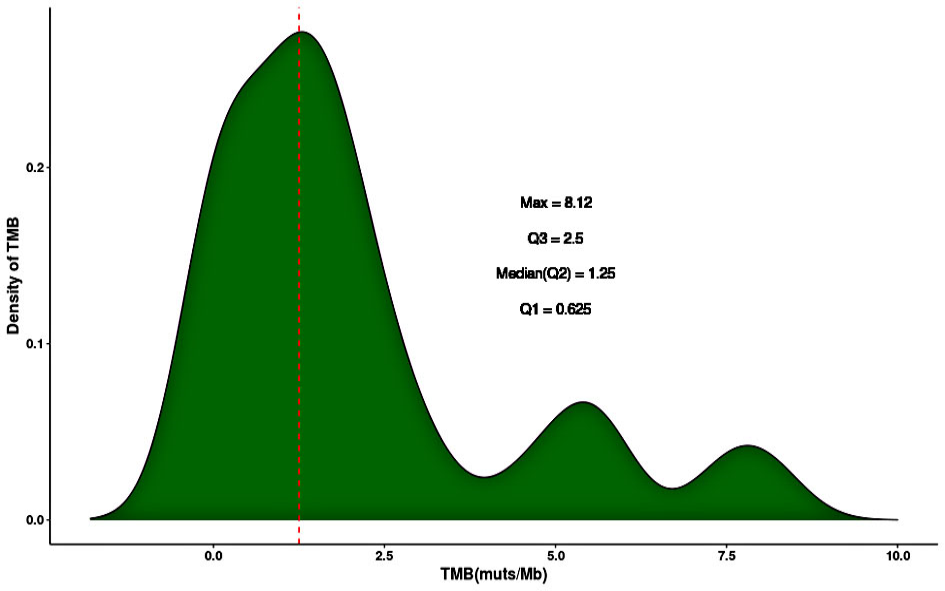
Distribution of TMB in 58 BC Tissue Samples. Density of TMB Represents the Percentage of BC Samples at Various TMB Levels. (Abbreviations: TMB, tumor mutation burden; muts/Mb, mutations/megabase; Max, maximum)

TMB value was calculated as the number of somatic, base substitution, non-silent, and indel mutations per coding area of the genomic sequence examines (~1.6 Mb). The TMB distribution in the 58 studied Egyptian BC patients was as follow: 19 cases in group A (33%), 12 cases in group B (21%), 14 cases in group C (24%), and 13 cases in group D (22%). The TMB value was between 0.00 and 8.12. The median value of TMB in the total BC cohort was 1.25 mutations/ Mb. The top quartile value and maximum value of TMB were 2.5 mutations/Mb and 8.12 mutations/Mb, respectively (Figure 1).

*TMB for different categories of the studied markers*

**Figure 2:**
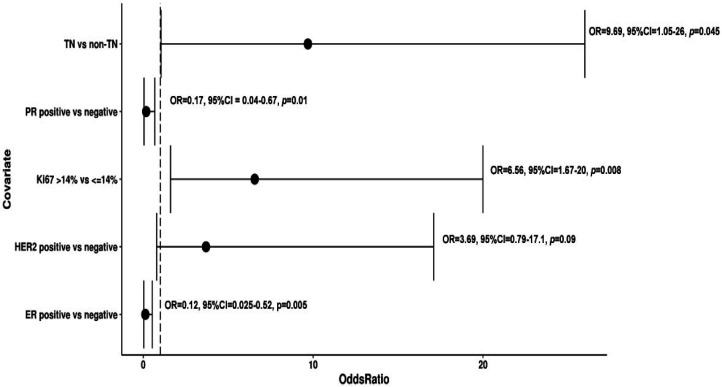
Forest Plot for Univariate Logistic Regression Analysis of TMB Distribution. (Abbreviations: OR, odds ratio; ER, estrogen receptor; PR, progesterone receptor; HER-2, human epidermal growth factor receptor 2)

Univariate logistic regression analysis of a training set with 10-fold cross validation showed that ER positive expression was significantly associated with TMB ≤ 1.25 (ER positive vs ER-negative, OR =0.12, 95% CI: 0.025–0.52, p= 0.005). Also, PR positive expression was significantly associated with TMB ≤1.25 (PR positive versus PR negative, OR = 0.17, 95% CI= 0.04-0.67, p= 0.01). On the other hand, Ki-67 expression positive was significantly associated with TMB >1.25 than those who were Ki-67 expression negative (Ki-67 positive versus Ki-67 negative, OR = 6.56, 95% CI= 1.67-20, p= 0.008). While, TMB >1.25 was significantly distributed in TN patients than non-TN patients (OR=9.69, 95% CI= 1.05-26, p=0.045). However, no significant differences were observed between HER2 positive and HER2 negative group (OR = 3.69, 95% CI: 0.79-17.1, p =0.09) (Figure 2).

*The distribution of ER/PR/HER2/Ki-67 in the four TMB groups*

We used the quartile method to measure the spread of values above and below the mean by dividing the distribution into four groups as it divides the dataset into three points; a lower quartile, median, and upper quartile. Table 2 shows TMB distribution in relation to patients’ demographic and clinical data. We found that ER and PR positivity associated significantly with lower TMB quartile while, higher TMB quartile was significantly observed in patients with positive Ki-67 expression and TNBC. Significant differences were found between positive and negative ER expression in TMB Group 1 (27.5% and 5.2%, respectively, p=0.0029) and TMB group 2 (18.9% and 1.7%, respectively, p=0.0038). Ki-67 index was significantly overexpressed in the top TMB quartile group 4 (20.1%) compared to the other groups (8.6%, p<0.001). The TNBC subtype associated significantly with the higher TMB group 4 compared to the other groups (p=0.025). For the distribution of PR, we observed that PR positivity was significantly higher in lower TMB group compared to the other groups (p= 0.017). A significant difference between HER2 positivity and negativity in the lower quartiles TMB group 1 (6.9% and 25.9%, respectively, p= 0.011) and group 2 (3.4% and 17.2%, p=0.02).

*Machine learning classification models for prediction of TMB in the studied BC patients*

**Figure 3:**
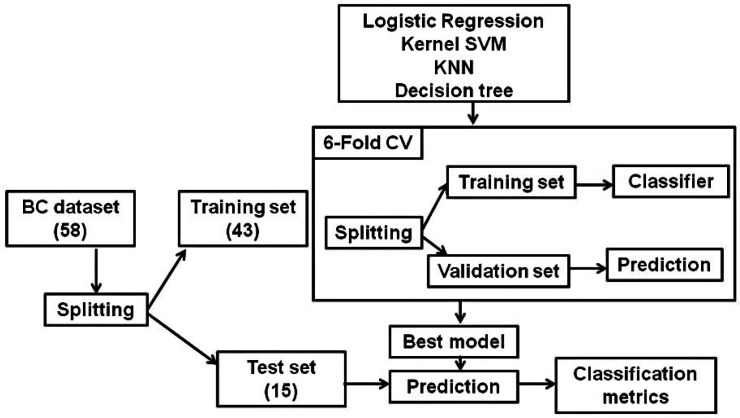
Pipeline Used to Develop Machine Learning Classification Model for Prediction of TMB in BC Cases. (Abbreviation: SVM, support vector machine; KNN, K-nearest neighbor)

Different classification machine learning algorithms; logistic regression, kernel support vector machine (SVM), K-nearest neighbor (KNN), Decision tree and Forrest decision, were initially evaluated to select the optimal classification model to predict TMB value according to patient’s characteristics (Figure 3). 

**Figure 4:**
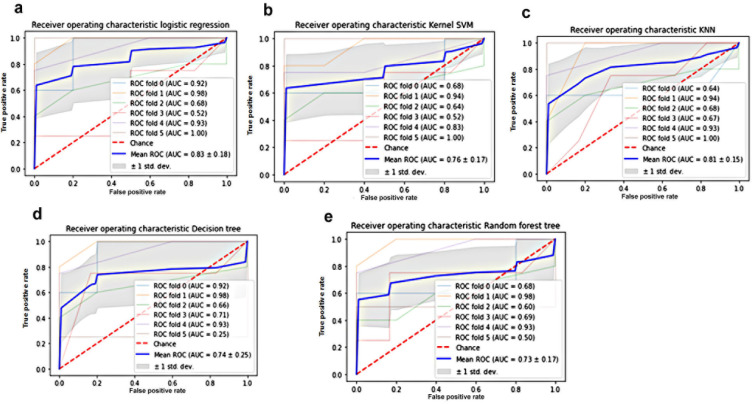
Classification Models Used for Prediction of TMB Value

The performance of the machine learning models with the best hyperparameters optimization was shown in Table 3. First, the most relevant features (ER, PR, HER2, Ki-67 and TN) were selected for prediction of TMB category using the Select KBest class of the scikit-learn library. The prediction of TMB was based on its categorization into 2 classes high TMB (>1.25 mutations/bp) and low TMB (≤1.25 mutations/bp). The KNN and decision tree were the best predictive model for prediction of TMB class based on the selected patient’s features having the highest accuracy (74.4 ± 9.76 and 74.11 ± 5.99, respectively) and AUC (0.81 ± 0.15 and 0.81 ± 0.25) (Figure 4). These were followed by logistic regression with an accuracy of 72.22 ± 6.4 and AUC of 0.83 ± 0.18 (Figure 4). The adjusted McFadden’s Pseudo R-Squared approach was used to evaluate the logistic regression model for prediction whether TMB ≤ median (group 1 and 2) or TMB > median (group 3 and 4). The adjusted Pseudo-R2 was 0.329 (P<0.001). The optimized logistic regression model was TMB = -27.5 -1.82 ER – 0.73 PR + 0.826 HER2 + 2.08 Ki-67 (Figure 4). 

**Table 1:** Clinical Features of the Studied 58 Egyptian BC Patients

Patients characteristics	Total (N =58)	Percentage (%)
Age(years)		
<55	27	46.6
≥55	31	53.4
Menopausal status		
Premenopausal	17	29.3
Postmenopausal	41	70.7
LN involvement		
0	10	17.2
1 to 3	17	29.3
>3	31	53.4
Grade		
1	4	6.9
2	45	77.5
3	9	15.6
ER		
Negative	20	34.5
Positive	38	65.5
PR		
Negative	26	44.6
Positive	32	55.2
HER2		
Negative	41	70.7
Positive	17	29.3
Ki67		
<14%	25	43.1
≥14%	33	56.9
Triple negative		
Non-TN	49	84.5
TN	9	15.5

**Table 2:** Quartile Group Distribution of TMB According to Clinicopathological Characteristics

Characteristic	Total (N)	TMB (%)				
		Group 1 (0.00-0.625) (n=19)	Group 2 (0.626-1.25) (n=12)	Group 3 (1.251-2.50) (n=14)	Group 4 >2.50 (n=13)	p value
ER						
Negative	20	3 (15.8)	1 (8.3)	8 (57.1)	8 (61.5)	0.003*
Positive	38	16 (84.2)	11 (91.7)	6 (42.9)	5 (38.5)	
PR						
Negative	26	4 (21.1)	4 (33.3)	9 (64.3)	9 (69.2)	0.017*
Positive	32	15 (78.9)	18 (66.7)	5 (35.7)	4 (30.8)	
HER2						
Negative	41	15 (78.9)	10 (83.3)	7 (50.0)	9 (69.2)	0.22
Positive	17	4 (21.1)	2 (16.7)	7 (50.0)	4 (30.8)	
Triple negative						
Non-TN	49	18 (94.7)	12 (100.0)	11 (78.6)	8 (61.5)	0.025*
TN	9	1 (5.3)	0 (0.0)	3 (21.4)	5 (38.5)	
Ki67						
<14%	25	14 (73.7)	6 (50.0)	4 (28.6)	1 (7.7)	0.0015*
≥14%	33	5 (26.3)	6 (50.0)	10 (71.4)	12 (92.3)	
Grade						
1	4	0 (0.0)	2 (16.7)	0 (0.0)	2 (15.4)	
2	45	17 (89.5)	6 (50.0)	12 (85.7)	10 (76.9)	0.12
3	9	2 (10.5)	4 (33.3)	2 (14.3)	1 (7.7)	
LN						
0	10	4 (21.1)	2 (16.7)	3 (21.4)	1 (7.7)	
1 to 3	17	4 (21.1)	3 (25.0)	3 (21.4)	7 (53.8)	0.52
>3	31	11 (57.9)	7 (58.3)	8 (57.2)	5 (38.5)	
Menopausal status						
Premenopausal	17	6 (31.5)	5 (41.7)	3 (21.4)	3 (23.1)	0.66
Postmenopausal	41	13 (68.5)	7 (58.3)	11 (78.6)	10 (76.9)	
Age						
<55	27	9 (47.4)	7 (58.3)	6 (42.9_	5 (38.5)	0.78
≥55	31	10 (52.6)	5 (41.7)	8 (57.1)	8 (61.5)	

**Table 3:** Performance Models Used for Prediction of TMB Level According to Patient’s Receptor Status

Model	Accuracy ± SD (%)	Mean AUC	Hyperparameters
Logistic Regression	72.22 ± 6.4	0.83 ± 0.18	penalty = L2 and C= 1.0
Kernel SVM	71.73 ± 12.04	0.76 ± 0.17	C = 1, kernel is Gaussian, and gamma is 0.01.
K Nearest neighbor (KNN)	74.4 ± 9.76	0.81 ± 0.15	Algorithm: auto, leaf_size: 1, n_jobs: -1, n_neighbors: 5
Decision tree	74.11 ± 5.99	0.81 ± 0.25	Criterion: gini , max_depth: 4 , Number Of Components: 3
Random forest tree	71.73 ± 12.04	0.73 ± 0.17	max_depth: 5, min_samples_leaf: 1, n_estimators: 25

## Discussion

BC is the most common malignancy among women worldwide. BC was classified based on the presence of the ER, PR, HER-2, and Ki-67, the routinely available markers in each BC specimen (Osako et al., 2017). The process of eliminating highly immunogenic tumor cells by somatic evolution is known as cancer immunoediting that uses the host immune system to protect it from tumor development. The increased burden of somatic mutations has been associated with the immunogenicity of BC (Criscitiello and Curigliano, 2015). Thus, we are in indeed need to assess the TMB to help in immunotherapy decision making.

In this study, the measured TMB was calculated by Ion AmpliSeq Comprehensive Cancer Panel using NGS technology. TMB value was between 0 and 8.13/Mb and majority of cases was distributed in the TMB group A (0.00-0.625) indicating the lower mutation rate in our studied patients. BC tumors have low immunogenicity to escape the immune recognition (Ross and Gay, 2017). Typically, BC harbors mutational loads averaging one mutation per Mb (Kandoth et al., 2013). Moreover, immune-suppressive factors are released in the tumor microenvironment which leads to difficult antigen presentation and results in low immune response (Mittendorf et al., 2007). Furthermore, cancer-associated immunogens are less common in BC (Disis et al., 1994). However, somatic mutations frequency or TMB is associated with the immunogenicity of BC (Criscitiello and Curigliano, 2015). Recently, TMB has emerged as a predictive marker to immunotherapy response across different tumors, including melanoma, lung cancer, and BC (Goodman et al., 2017). However, the heterogeneity of TMB among BC subtypes is not well characterized.

Here in, we described the TMB differences between hormone receptor-positive and hormone receptor-negative Egyptian BC patients. Our findings showed that hormone receptor-positive was associated with TMB ≤1.25 (ER positive vs ER-negative, OR =0.35, 95% CI: 0.04–2.98, p= 0.001) (PR positive versus PR negative, OR = 0.17, 95% CI= 0.02-0.44, p= 0.002), which comes in agreement with a recent study by Xu et al., (2019). 

Matching with Xu et al., (2018), we revealed that higher TMB was distributed in Ki-67 expression positive patients than those with Ki-67 expression negative. However, they reported that patients with HER-2 expression positive was significantly associated with TMB (HER-2 positive vs HER-2 negative, OR =34.81, 95% CI: 3.711–821.689, P=0.0065) which was not the situation in our study (Xu et al., 2018). In the current study, no significant differences of TMB were observed between HER2 positive and HER2 negative groups (OR = 2.47, 95% CI: 0.64-9.54, p =0.19). Based on the current findings, we suggest that hormone receptor-negative BC or Ki-67 expression positive BC exhibits relatively elevated TMB and immunotherapeutic options are recommended.

Many ongoing immunotherapeutic approaches in BC are used to increase the quality or quantity of effector immune cells, and eliminate cancer-induced immunosuppression. For example, therapeutic administration of monoclonal antibodies to treat metastatic TNBC, including CTLA-4, PD-1 or Treg cells (Adams et al., 2019). In the present study, our findings suggested that hormone receptor positive and Ki-67 expression positive Egyptian BC patients showed high immunogenic activity when compared to hormone receptor negative and Ki-67 expression negative patients. Thus, they may be considered for immune checkpoint inhibition and suggesting the use of TMB as a biomarker for cancer immunotherapy.

Not only immunogenic neoantigens, but also mutations in immunologically relevant genes, can affect the ability of T cells to identify and kill tumor cell, for example; mutations in JAK1, JAK2, B2M, and STK11genes (Skoulidis et al., 2018). We previously reported the presence of certain somatic mutations in JAK2, JAK3, and STK11 genes in a cohort of Egyptian BC patients (Nassar et al., 2020). Hence, we suggest conducting targeted sequencing of immunologically relevant genes in addition to TMB to be used as biomarkers to enhance predicting the patients’ prognosis and response to immunotherapy.

In conclusion, we showed that hormone receptor negative and Ki-67 expression positive Egyptian BC patients exhibit TMB value >1.25. We also showed that TMB value can be predicted based on the expression level of ER, PR, HER-2, and Ki-67 and the optimized logistic regression model was TMB = -27.5 -1.82 ER – 0.73 PR + 0.826 HER2 + 2.08 Ki-67. 

## Author Contribution Statement

Abdel-Rahman N Zekri designed the study. Ahmed M. Lymona, Tamer M. Manie recruited patients and collected clinical data. Auhood Nassar, Ahmed M. Lymona, Mai M. Lotfy, and Amira Salah El-Din Youssef performed library preparation and NGS workflow. Marwa Mohanad performed the statistical analysis. Auhood Nassar drafted the manuscript. Mina M G Youssef and Iman G. Farahat revised the manuscript. All authors read and approved the final version.

## Recommendation

We recommend increasing sample size to avoid false positive results. Also, following up the patients until recurrence or metastasis is crucial to find the correlation between TMB and the survival data.
